# New perspectives in the echocardiographic hemodynamics multiparametric assessment of patients with heart failure

**DOI:** 10.1007/s10741-024-10398-7

**Published:** 2024-03-20

**Authors:** Matteo Lisi, Giovanni Andrea Luisi, Maria Concetta Pastore, Giulia Elena Mandoli, Giovanni Benfari, Federica Ilardi, Alessandro Malagoli, Simona Sperlongano, Michael Y. Henein, Matteo Cameli, Antonello D’Andrea

**Affiliations:** 1 Department of Cardiovascular Disease–AUSL Romagna, Division of Cardiology, Ospedale S. Maria delle Croci, Viale Randi 5, 48121 Ravenna, Italy; 2 Department of Medical Biotechnologies, Division of Cardiology, University of Siena, Siena, Italy; 3 Section of Cardiology, Department of Medicine, University of Verona, Verona, Italy; 4 Department of Advanced Biomedical Sciences, Division of Cardiology, Federico II University Hospital, Via S. Pansini 5, 80131 Naples, Italy; 5 Mediterranea Cardiocentro, 80122 Naples, Italy; 6 Division of Cardiology, Nephro-Cardiovascular Department, Baggiovara Hospital, Baggiovara, Italy; 7 Division of Cardiology, Department of Translational Medical Sciences, University of Campania Luigi Vanvitelli, Naples, Italy; 8 Department of Public Health and Clinical Medicine, Umeå University, Umeå, Sweden; 9 Department of Cardiology, Umberto I Hospital, 84014 Nocera Inferiore, SA Italy

**Keywords:** Heart failure, Stroke volume, Hemodynamics, Echocardiography, Diagnosis, Prognosis

## Abstract

International Guidelines consider left ventricular ejection fraction (LVEF) as an important parameter to categorize patients with heart failure (HF) and to define recommended treatments in clinical practice. However, LVEF has some technical and clinical limitations, being derived from geometric assumptions and is unable to evaluate intrinsic myocardial function and LV filling pressure (LVFP). Moreover, it has been shown to fail to predict clinical outcome in patients with end-stage HF. The analysis of LV antegrade flow derived from pulsed-wave Doppler (stroke volume index, stroke distance, cardiac output, and cardiac index) and non-invasive evaluation of LVFP have demonstrated some advantages and prognostic implications in HF patients. Speckle tracking echocardiography (STE) is able to unmask intrinsic myocardial systolic dysfunction in HF patients, particularly in those with LV preserved EF, hence allowing analysis of LV, right ventricular and left atrial (LA) intrinsic myocardial function (global peak atrial LS, (PALS)). Global PALS has been proven a reliable index of LVFP which could fill the gaps “gray zone” in the previous Guidelines algorithm for the assessment of LV diastolic dysfunction and LVFP, being added to the latest European Association of Cardiovascular Imaging Consensus document for the use of multimodality imaging in evaluating HFpEF. The aim of this review is to highlight the importance of the hemodynamics multiparametric approach of assessing myocardial function (from LVFP to stroke volume) in patients with HF, thus overcoming the limitations of LVEF.

## Introduction

Left ventricular ejection fraction (LVEF) is clinically considered a key parameter in the evaluation of patients with heart failure (HF) [1]. International Guidelines categorized HF on the basis of LV EF, classifying patients into three groups: those with preserved (HFpEF, LVEF ≥ 50%); mildly reduced (HFmrEF, LVEF 41–49%); and reduced LVEF (HFrEF, LVEF ≤ 40%) [2]. This HF categorization is based on inclusion criteria of clinical trials and registers and is used by current European Society of Cardiology (ESC) Guidelines when defining recommended treatments in clinical practice [3]. The use of LVEF has certainly many advantages; being a universally accepted and easily calculated index of cardiac function, using basic echocardiography [4]. However, LVEF has some limitations. Firstly, there is no relationship between LVEF and symptoms, and secondly, it is unable to predict clinical outcome in patients with end-stage HF. These limitations are because of the fact that EF only reflects geometric changes of LV rather than the intrinsic contractile function of the myocardium and LV filling pressures (LVFP) [5]. The analysis of LV myocardial deformation obtained by speckle tracking echocardiography (STE) could unmask hidden systolic dysfunction in all categories of LVEF [6]. In patients with preserved LVEF, a wide range of global longitudinal strain (GLS) disturbances have been shown, suggesting many categories of disease severity [7]. The only group of HF patients in whom LVEF is able to stratify prognosis is HFrEF, but not HFpEF or HFmrEF [8].

HF is a progressive condition with a clinical picture resulting from reduced forward flow and/or elevated LVFP in which abnormal myocardium function is responsible for the failure of the heart to pump blood at a rate compatible with the requirements of the tissues during ordinary activity [9]. This condition causes a reduced cardiac output (CO) and/or elevated LVFP, causing the classical clinical signs and symptoms of HF.

Although assessment of cardiac hemodynamics is important in the prognostic stratification and management of patients with HF, the need for an invasive method limits its application in clinical practice because of the small but definite risk of infections, bleeding, and pneumothorax and the discomfort and the cost of the procedure. To date, Doppler echocardiography allows us to obtain valuable measures of both output indices and LVFP [10–12]. Although the cardiac ultrasound technique is widely available, non-invasive, and easily repeatable, the assessment of LVFP and outflow variables by echo-Doppler has been hampered by difficulties in obtaining estimates that could have value in a variety of cardiac disorders.

The aim of this review is to highlight the importance of the hemodynamics multiparametric approach of assessing myocardial function (from LVFP to stroke volume) and new perspectives and recent developments for a comprehensive non-invasive evaluation in patients with HF, thus overcoming the limitations of LVEF.

## Echocardiographic evaluation of left ventricle antegrade flow

**Fig. 1:**
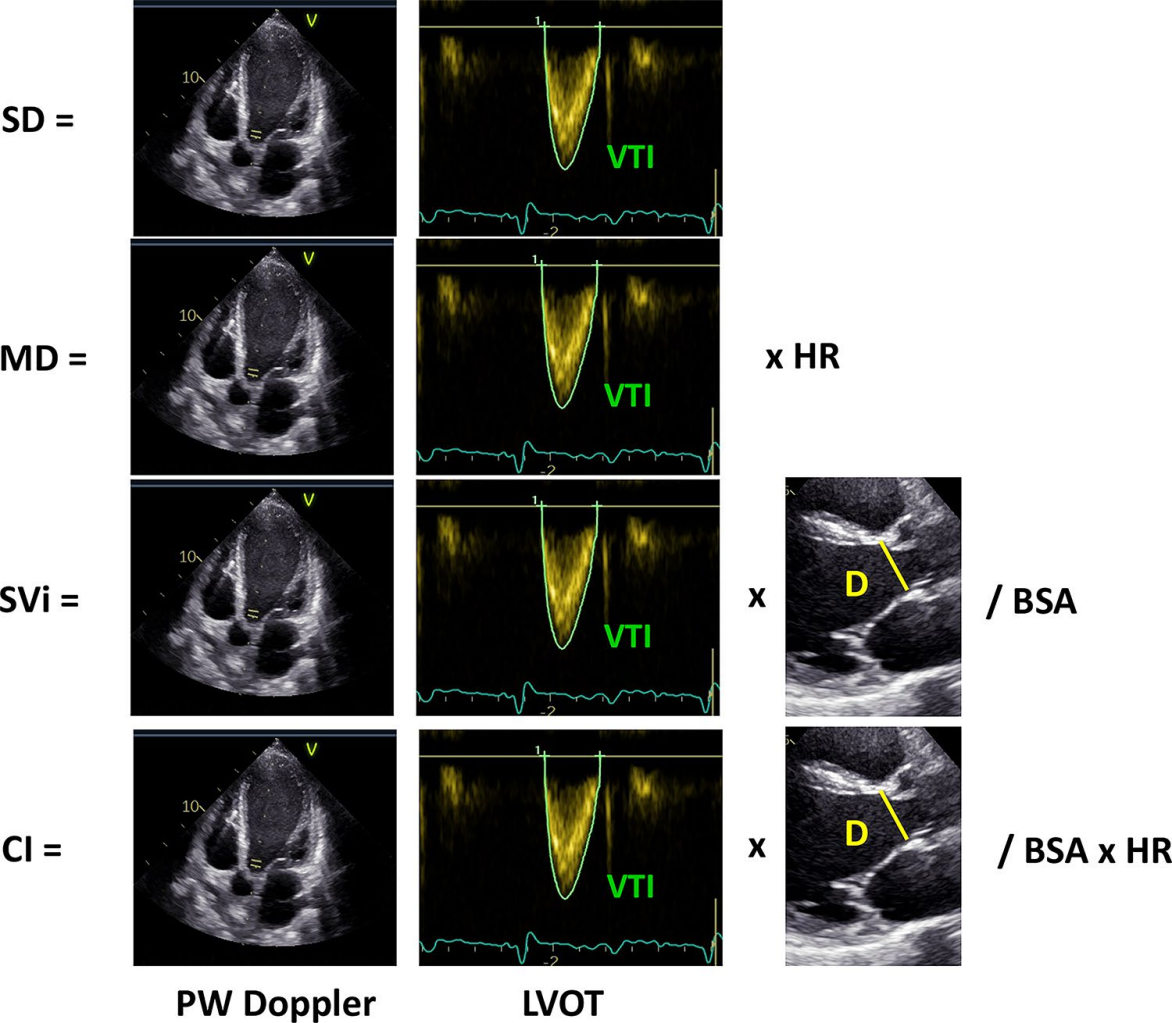
Echocardiographic evaluation of left ventricular blood ejection from the apical 5-chamber view

LV antegrade flow can be expressed as cardiac output (CO, l/min), calculated from stroke volume (SV), or cardiac index normalized for body surface area (CI, l/min/m^2^), which are measures of blood flow ejected by LV per minute [13]. SV or SV normalized for body surface area (SVi) represents the volume of blood ejected by the LV in a single beat. The most widely accepted thresholds for normal cardiac index and SVi are ≥ 2.0 l/min/m^2^ [14] and ≥ 30 ml/m^2^ [15]. These parameters are derived from the stroke distance (SD, cm) and minute distance (MD, m/min), obtained by integrating the velocity-time curve of pulsed-wave Doppler at the LV outflow tract (LVOT) level, recorded from the apical 5-chamber view (Fig. 1). Doppler-derived SV can be assessed as the product of the LVOT cross-sectional area and the LVOT time-velocity integral (VTI) obtained by pulsed-Doppler. CO can be measured as the product of SV and heart rate. SV index (SVi) and CI can be derived by normalizing SV and CO for BSA [4]. Therefore, outflow parameters can be divided into two groups: per-beat (SD, SV, SVi) and per-minute (MD, CO, CI). Per-minute parameters have some limitations, in particular the compensatory effect of tachycardia that could result in normal values despite reduced SV/SVi [4]. On the contrary, per-minute measurements seem preferable in circumstances in which an increased HR ensures the maintenance of antegrade flow and organ perfusion. Table 1 summarizes per-beat and per-minute outflow parameters, including relative advantages and disadvantages. Previous studies have validated the Doppler method for the calculation of SV, showing excellent feasibility and good correlations between invasive and Doppler measurements of SV and CO [16–18] and others have also validated non-invasive Doppler estimation of SV. These studies reported a good correlation between echocardiographic SV and CO calculated by thermodilution [19] or the Fick method [16], independent of tricuspid regurgitation severity [20].

**Table 1:** The per-beat and per-minutes outflow parameters: advantages and disadvantages

		**Advantage**	**Disadvantage**
**Per-beat**	**SD**	Easy to calculate Independent from LVOT area Represent LV contraction The most accurate in predicting mortality if ≤ 18 cm	Unreliable if flow acceleration in LVOT (ex. obstructive CMPI) Reduced by high FC Influenced by valve regurgitation
	**SV**	Represent volume ejected by LV in each beat	LVOT area depend by BSA Error in estimation of LVOT
	**SVi**	Represent if the volume ejected is adequate for BSA Normal: > 30 ml/m2 Prognostic implication	LVOT area depend by BSA Error in estimation of LVOT Over/underestimation in extreme weight
**Per-minute**	**MD**	Easy to calculate Independent from LVOT area Take into account HR	Could be normal if high compensatory HR Unreliable if flow acceleration in LVOT (ex. obstructive CMPI) Influenced by valve regurgitation
	**CO**	Represent the volume ejected by LV in one minute	Depend from LVOT area Influenced by HR No correlation with outcome
	**CI**	Represent if the volume ejected in one minute is adequate for BSA Normal: > 2.0 l/min/m2	Depend from LVOT area No correlation with outcome Over/underestimation in extreme weight

## Echocardiographic evaluation of heart failure based on hemodynamics approach

The diagnosis of chronic HF requires clinical evaluation of symptoms and/or signs of HF [2]. However, the 2021 HF ESC Guidelines stress that symptoms and signs lack sufficient accuracy to be used alone to make the diagnosis of HF, and objective evidence of cardiac dysfunction based on brain natriuretic peptide (BNP), electrocardiogram (ECG), and echocardiography is required [2]. Echocardiography is recommended to evaluate LVEF (also for classification of HF), chamber size, presence of LV hypertrophy, regional wall motion abnormalities, valve dysfunction, and pulmonary hypertension [2]. The ESC Guidelines also recommend evaluation of echocardiography diastolic function for diagnosis of HF [2], but evaluation of cardiac output is not considered, neither for diagnosis nor for outcome. However, Doppler assessment of LVFP and cardiac output correlate with symptoms better than LVEF because they reflect the hemodynamic alteration of HF [20]. In HF patients, addition of echocardiographic markers of LVFP to clinical assessment resulted in improved reclassification by 1.5 times compared with only clinical assessment [21].

### Echocardiographic assessment of left ventricular filling pressure and its prognostic impact

Cardiac catheterization is the gold standard investigation for direct measurement of LVFP but is not practical for widespread application or serial longitudinal follow-up examinations [12]. Doppler echocardiography has become well accepted as a reliable, reproducible, and practical non-invasive method for diagnosis and longitudinal follow-up of patients with diastolic dysfunction [12]. Grading of diastolic dysfunction reflects different stages of cardiac myocardium disease: with disease progression, left atrial (LA) pressure increases, thus increasing the driving pressure across the mitral valve and consequent increase in the E velocity on the mitral flow velocity curve and a restrictive pattern appear in the late stage of myocardial disease [12].

Echocardiographic indices of LVFP correlate with invasive estimation by catheterization. Transmitral E/A ratio, average E/e′ ratio, and LA volume index (LAVi) were independently associated with invasive LV diastolic pressure (LVEDP) [22, 23]. In the Euro-Filling study, there was a different correlation of echocardiographic indices of LVFP with invasive estimation among patients with different LVEF [22]: E/e′ lateral was significantly related to LVEDP in patients with preserved LVEF while E/A ratio best correlated with invasive LVEDP in patients with reduced LVEF [22]. However, in a meta-analysis involving 3540 patients, the restrictive mitral filling pattern proved a powerful predictor of mortality, independent of LVEF, age, and etiology [24].

Echocardiographic estimation of LVFP has a prognostic impact. In patients with dilated cardiomyopathy, mortality plus heart transplantation were significantly higher in patient with restrictive LV filling pattern (diagnosed when E wave deceleration time (EDT) < 115 ms) than patients with non-restrictive filling and the persistence of restrictive filling at 3 months is associated with a high rate of primary endpoint [25]. Also, a short (≤ 125 ms) deceleration time by mitral Doppler adds important prognostic information compared with other clinical, functional in both symptomatic and asymptomatic patients [26].

Assessment of Doppler pulmonary venous flow has also been shown to have a role in the prognostic evaluation of patients with LV dysfunction: the difference between duration of mitral A wave and pulmonary vein atrial reversal flow ≥ 30 ms provided important prognostic information with regard to cardiac mortality and emerged as the single best predictor of cardiac events (cardiac mortality, hospitalization) [27].

It is well known that there is a correlation between pulmonary regurgitation end-diastolic pressure gradient (PRG) and pulmonary artery diastolic pressure (PADP) [28]. Also, the latter correlates with pulmonary artery wedge pressure (PAWP) [29]. Echocardiography estimation of PADP (e-PADP) were calculated as the sum of PRG added to the estimated right atrial pressure (RAP) [30]. In patients with cardiac disease without pre-capillary pulmonary hypertension, an algorithm based on e-PADP has been shown to accurately estimate elevated LVFP independent of LVEF, providing an advantage in accuracy over the American Society of Echocardiography (ASE)/European Association of Cardiovascular Imaging (EACVI) 2016 recommendations [30].

### The contributions and limitations of international Guidelines for the assessment of raised left ventricular filling pressure and the role of left atrial strain

**Fig. 2:**
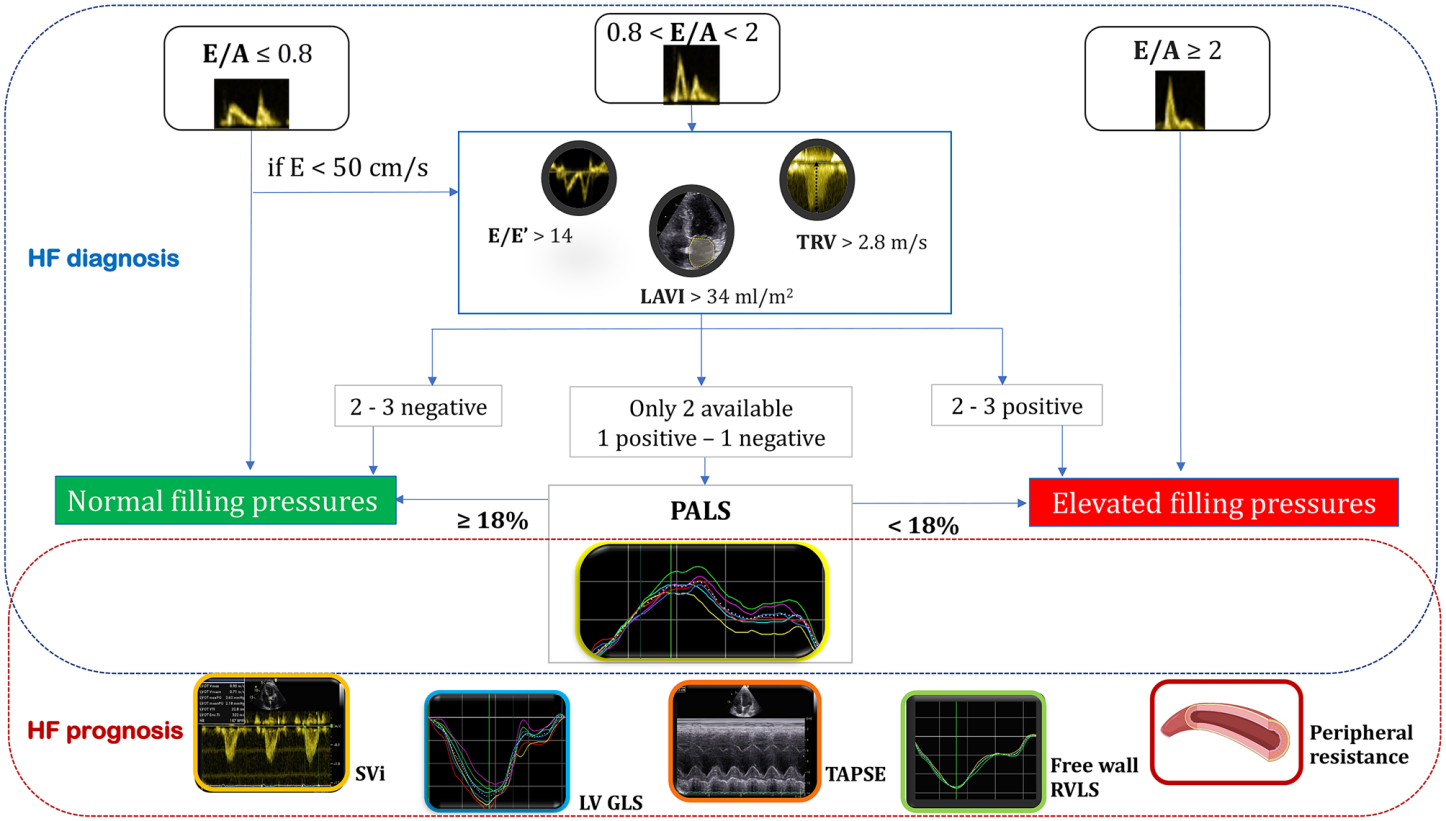
The key role of global peak atrial longitudinal strain

The 2016 ASE/ EACVI Guidelines on assessment of LV diastolic dysfunction [31] established two algorithms for LVFP estimation, combining LA volume index with Doppler velocities (mitral inflow: pulsed wave Doppler; tissue Doppler at lateral and medial mitral anulus; tricuspid peak velocity): an algorithm for normal LVEF and another for reduced LVEF or for patients with normal LVEF but with myocardial disease after consideration of clinical and other 2D data [31]. The Guidelines’ algorithm was validated [21] and proved to have 87% overall accuracy in detecting elevated PCWP (evaluated invasively) which made it applicable in 419 (93.1%) patients and was inconclusive in 31 (6.9%) patients. This approach has also good accuracy in patients with left bundle branch block or paced rhythm (area under the curve (AUC) = 0.84), atrial fibrillation (AF; AUC = 0.83), or moderately severe to severe mitral regurgitation (AUC = 0.96) [21]. Other authors [22] found that the 2016 algorithm of diastolic dysfunction was superior to the 2009 recommendations in estimating invasively calculated LV diastolic pressures. However, this algorithm presents a “gray zone” of indeterminate diastolic function. It is estimated that in 10% of patients, evaluation of LVFP (according to these Guidelines) [31] is inconclusive due to missing echocardiographic parameters, most often TR velocity [32]. For these cases, the 2016 EACVI/ASE Guidelines [31] suggest the use of supplementary parameters such as pulmonary vein velocities, isovolumetric relaxation time, or other methods, such as STE. Particularly, in the last years, LA strain (global peak atrial longitudinal strain (PALS), Fig. 2) by STE has been proved a reliable index of LVFP which could fill the gaps in the previous algorithm [33].

LA strain has been described as a more sensitive parameter than LA volume for early detection of LA structural and functional impairment. In the early phase of LV diastolic dysfunction, LA volume can still be normal, but the progressive and chronic increase of LVFP leads to LA ultrastructural abnormalities, which are detected by reduced LA strain as reduced compliance, and LA dilatation which occurs in the last phase [34]. Considering the thin wall of LA, cavity deformation mechanics (PALS) are impaired in patients with hypertension or diabetes despite normal LVEF even when LA indexed volume is normal, in a pre-clinical phase; the coexistence of both conditions further impairs LA performance in an additive fashion [35]. LA strain offers a quick analysis of cavity deformation and is correlated with invasively assessed LVFP [36]. It also provides additive value for diastolic function classification [37, 38] and the HFpEF diagnosis [39, 40]. Indeed, in the latest EACVI consensus document for the use of multimodality imaging in evaluating HFpEF, the use of LA strain is suggested in case of missing parameters with only two criteria available, one positive and another negative, with the cut-off value of LA reservoir strain (PALS) < 18% to define elevated LVFP [41].

A recent multicenter study of 322 patients referred for diagnostic right- or left-side heart catheterization and with a mean LVEF of 55%, demonstrated that both PALS and pump strain (peak atrial contraction strain (PACS)) were associated with LVFP, with an optimal cut-off to differentiate between normal and elevated LVFP (the latter defined as PCWP > 12 mmHg) of 18% for PALS and 8% for PACS [32].

Similar results were also confirmed [42] in 210 patients with LVEF > 50% (comparing echocardiography and right heart catheterization), showing that PALS displayed a strong ability to identify patients with elevated LVFP (PCWP > 15 mmHg) with an AUC = 0.76. Moreover, substituting TR peak velocity for PALS (cut-off value of < 18%) in the 2016 ASE/EACVI algorithm resulted in 91% feasibility, 81% accuracy, and stronger agreement with invasive measurements. These results confirm that HFpEF is a challenging diagnosis, requiring an integrated approach (combination of multiple echocardiographic parameters, standard diagnostic test, and physical examination), and they also show that LA strain is superior to TR velocity as a marker of LVFP, very useful to include in the diagnostic algorithm of patients with symptoms of HF and normal LVEF [43]. A correct measurement of TR peak velocity is not so feasible in the clinical practice, and two recent studies have shown that TR was available only in 40–60% of patients [22, 44], thus becoming a problem especially in patients with suspected HFpEF.

Interestingly, the latest EACVI algorithm for the use of a multimodality imaging approach to evaluate HFpEF can also be used in HFrEF in sinus rhythm [41], allowing echocardiographic estimation of LVFP in patients with HF, regardless of LVEF value.

It has recently been demonstrated that in patients with advanced HF, undergoing heart transplantation, the global PALS was inversely correlated with PCWP (*R* =  − 0.83; *p* < 0.0001) and with LV fibrosis severity (*R* =  − 0.78; *p* < 0.0001) but did not correlate with LVEF (*R* = 0.15; *p* = 0.2); moreover among echocardiographic indices of LVFP, global PALS proved the strongest [AUC 0.955 (95% CI 0.87–0.99)] predictor of raised (> 18 mmHg) PCWP, evaluated invasively [45].

These results highlight the fact that LA myocardial dysfunction is strongly correlated with PCWP, confirming previous results and showing that global PALS has added value in the non-invasive assessment of LVFP in HF patients, irrespective of LVEF [46].

However, some limitation must be considered. In patients with cardiac resynchronization therapy (CRT), left bundle branch block, and RV pacing the algorithm has less accuracy. In patients with hypertrophic cardiomyopathy, more than moderate mitral regurgitation, mitral stenosis, mitral annular calcification, mitral valve repair/prosthetic mitral valve, LV assist device, and high output HF the same algorithm should not be applied [41]. It is also estimated that in approximately 50% of patients with HFpEF, the LV GLS values are normal at rest and a high percentage of patients develop symptoms of HF only during exercise, consequentially resting echocardiographic values may not be sufficient, hence making exercise echocardiographic measurements crucial (diastolic stress test) [41, 47]. The EACVI/ASE recommend a stepped protocol, starting at 25 W at 60 r.p.m. with the load increasing by 25 W every 3 min until the patient has reached maximal predicted workload and/or maximal predicted HR (220, age in years) and/or developed limiting symptoms [48]. Exercise echocardiography should be considered abnormal if the average E/e′ ratio at peak stress increases to ≥ 15, with or without a peak TR velocity > 3.4 m/s [31, 48, 49].

### Echocardiographic evaluation of compromised forward outflow and its prognostic impact

In many studies, SD was the most accurate prognostic predictor in HF, with a cut off of 22 cm [50] or 18 cm [20] (the cut off value depends on the disease status and the context (hospitalized or ambulatory) in which patients have been evaluated instead of CI which is not associated with mortality, probably because of the compensatory high HR during low output state [51]. In outpatients with coronary artery disease, a SD < 22 cm predicts HF hospitalization [50] and the lowest is SD, and the worst is the prognosis of patients (SD < 18 cm predicts the combined end point of HF hospitalization or mortality) [20]. In a cohort of patients with HF and extremely low LVOT VTI (cut off 10 cm) at baseline, the lowest tertile cut-off of 8 cm strongly predicts an adverse outcome (combination of 12-month death and LV assist device implantation) [52].

In a population of patients hospitalized due to HF, SD was independently associated with 5-year all-cause mortality, while LVEF was not and patients with stroke distance below 15,7 have 82% higher age-adjusted risk for death during follow-up [51]. A recent study showed that LV output evaluated as per-beat index, such as SVi (< 30 mL/m^2^), as a better predictor of outcome than CI (per-minute index) [53]. In HF patients, the degree of diastolic dysfunction was a stronger predictor of mortality than LVEF [54] and a high E/e′ ratio at 1-year follow-up predicted poor long-term outcome [55]. Some authors [15] tried to integrate CO and LVFP echocardiographic indices all together, to validate a prognostic model and subdivided patients hospitalized for HF into four categories based on SVi (< 30 mL/m^2^), LVFP estimation (using the validated ESC Guidelines algorithm) [56] and the presence of RV dysfunction (RVD, defined as TAPSE < 17 mm): normal flow-normal pressure (NF-NP), normal flow-high pressure (NF-HP), low flow (LF) with no RVD (LF-NRVD), LF with RVD (LF-RVD) [15]. This approach, based on echo-directed hemodynamics profiles, predicted the clinical outcome of hospitalized patients with HF and proved superior to LVEF in prognostic stratification. The LF status identified patients with a worse prognosis, in particular, the LF-RVD group had a major number of adverse events and NF-NP had the better prognosis [15].

We should underline the useful combination of different echocardiographic parameters of either diastolic dysfunction or reduced outflow, during follow up of patients with HF [14],[57). The combination of these parameters outperforms EF in both the assessment and the outcome prediction of patients with HFrEF [14, 57].

## Left ventricular myocardial longitudinal strain: a useful tool in hemodynamics evaluation of HF patients

LV GLS by STE allows evaluation of LV myocardial function in patients with HF [5]. GLS is superior to LVEF and other longitudinal markers (such as tissue Doppler imaging) in identifying HF patients with poor clinical outcome [58]. Some authors [57] divided HF patients in four hemodynamics phenotypes based on LV SVi, LVFP, and right ventricular (RV) function: normal output-normal LVFP (NO-NP), normal output-high LVFP (NO-HP), low output- no RV dysfunction (LO-NRVD), and low output-RV dysfunction (LO-RVD). LV GLS of these patients showed progressive impairment of hemodynamics phenotypes. The highest prognostic value added by LV GLS was in patients with normal SVi and it was not associated with a prognostic endpoint in the LO subgroups, GLS <  − 12% identified patients with the worst prognosis in the NO-NP and NO-HP [57].

## New directions in the assessment of hemodynamics variables

The hemodynamics study of HF patients involves a multiparametric approach. The estimation of systemic vascular resistances (SVR) turned out to be important, considering its role in maintaining systemic blood pressure and organ perfusion, particularly during reduction of SV in HF patients [59].

ASE Guidelines support echocardiography estimation of SVR [60] in a critical setting. This approach was recently validated in a prospective comparison study assessing the SVR index by both echocardiography and transpulmonary thermodilution in 28 patients hospitalized for cardiogenic shock, on admission and after treatment [61]. The authors observed a good correlation between invasive and echocardiographic measures of estimation of SVR (*r* = 0.86, 95% confidence interval 0.74, 0.93; *p* < 0.0001) [61].

The SVR index (SVRi) [60] is determined using mean arm arterial blood pressure (MAP), right arterial pressure (RAP), and cardiac index with the following formula: SVRi (dynes. s. m^2^/cm^−5^) = (MAP – RAP) (mmHg) × 80/cardiac index (L. m^−1^. m^−2^). RAP was estimated on the basis of inferior vena cava size and its breathing-related collapsibility [60]: size ⩽2.1 cm and collapses > 50% during sniff = RAP 0–5 mmHg; size > 2.1 cm and collapses > 50% during sniff = RAP 5–10 mmHg; size > 2.1 cm and collapses < 50% during sniff = RAP 10–20 mmHg. Another simplified method for estimating SVR has been validated, using the ratio of the peak mitral regurgitant velocity (MRV) (m/s) to LVOT VTI (cm) by Doppler echocardiography [62]. This parameter correlated better with invasive right heart catheterization estimation of SVR. It has been demonstrated [63] that the prognostic value of the estimated SVR analogue (eSVR), calculated as the ratio of systolic blood pressure to LVOT VTI is associated with a higher risk of adverse outcomes, including HF, MACE, and all-cause mortality. The highest eSVR tertile (≥ 6.9) had the highest risk of adverse events compared to the lowest tertile (< 5.6) that was associated with the best outcome [63]. eSVR is easier to calculate, without a need to calculate MAP, the CO, or RAP.

Estimation of pulmonary vascular resistance (PVR) is also useful, in the prospect of performing a complete non-invasive hemodynamics evaluation. The Doppler-derived equation for estimating PVR (PVR = PAPm_echo_ − PCWP/CO_echo_, where PAPm_echo_ is echocardiographic estimation of mean pulmonary arterial pressure and PCWP is assumed 10 mmHg) has proved very accurate in identifying patients with raised PVR with strong correlation (*r* = 0.87, *p* < 0.001) with the respective catheter-based measurements [64].

Accordingly, there is not a single parameter that is unique in evaluating patients with HF, in particular those with EFpEF, making it necessary to combine several parameters to accurately establish etiology (such as hypertensive cardiomyopathy or chronic coronary disease) and to grade diastolic dysfunction; to measure LV and LA myocardial intrinsic function (strain), LA volume index, SVi; and to estimate LVFP. In HFrEF patients, the LV antegrade flow is expected to be low (as shown by reduced SVi). In contrast, it is not so obvious in patients with HFpEF in whom there is a wide spectrum of patients with a LF “paradoxical” phenotype [65]. In a cohort of stable outpatients with HFpEF, it has been demonstrated [66] that 37% had a LF phenotype, using a SVi cut-off value of < 35 ml/m^2^. Some recently [65] reported lower (23%) LF, using a SVi cut-off value of < 30 ml/m^2^, in hospitalized HFpEF patients which were better associated with outcome, compared to SVI < 35 ml/m^2^, as previously described by the same group [53]. Patients with HFpEF and LF phenotype were associated with smaller LV cavity size (LV end-diastolic diameter indexed [EDDi], measured at the level of the mitral valve leaflet tips: EDDI < 25 mm/m^2^ in males and < 26 mm/m^2^ in females) with LV concentric remodeling, RV dysfunction (defined as tricuspid annular plane systolic excursion/systolic pulmonary artery pressure ratio [TAPSE/sPAP] of < 0.36 mm/mmHg, as previously identified)[67] and AF at the time of echocardiographic evaluation [65]. Chronic pressure overload due to arterial hypertension (AH) is the typical pathophysiological model which if not properly treated can lead to HEpEF. Approximately 20–60% of patients with uncomplicated AH have echocardiographic evidence for increased LV mass (and stiff chamber) [68], caused by increased wall thickness and concentric hypertrophy which over time (in complicated AH) can determine increased LV and LA filling pressure, diastolic dysfunction and myocardial fibrosis, eventually evolving to AF [69] and congestive HF if untreated [5].

In HFpEF, LA plays a key role in preserving good LV function and the asymptomatic status of the patients [70] since the very first stage of diastolic dysfunction contributes in maintaining normal cardiac filling and output [71], before major remodeling occurs, which alters cardiomyocytes and interstitial ultrastructure, leading to LA myocardial fibrosis [72]. Eventually, the latter determines high LVFP [73], until the development of HFrEF [74]. Late-stage HF patients involve RV enlargement and reduced systolic function (in the form of reduced RV free-wall longitudinal strain) due to significant RV myocardial fibrosis [75], determining poorer exercise capacity [76, 77] and reduced survival [78, 79].

## Conclusion

Currently, Doppler echocardiographic estimates of hemodynamics variables are accurate and reproducible and can provide a thorough hemodynamics evaluation of HF patients independent of the type of HF, also in advanced HF. The available evidence supports an integrated approach to HF categorization based on cardiac hemodynamics (LV systolic forward flow, LVFP), in association with LV and LA myocardial strain and RV systolic function in order to better ascertain the patient’s pathophysiology and stratify prognosis. We therefore suggest overcoming the limitation of measuring LVEF by incorporating the above-discussed modalities with their documented advantages.

## Data Availability

Not applicable.
